# Real‐World longitudinal practice patterns in the use of PD‐1 and PD‐L1 inhibitors as First‐Line therapy in patients with Non‐Small cell lung cancer in the United States

**DOI:** 10.1002/cam4.4785

**Published:** 2022-05-02

**Authors:** Rajwanth Veluswamy, Fred R. Hirsch, Emanuela Taioli, Juan Wisnivesky, Ross Strauss, Douglas Harrough, Boxiong Tang, Gisoo Barnes

**Affiliations:** ^1^ Tisch Cancer Institute Icahn School of Medicine at Mount Sinai New York City New York USA; ^2^ Center for Thoracic Oncology Tisch Cancer Institute and Icahn School of Medicine at Mount Sinai New York City New York USA; ^3^ Institute for Translational Epidemiology Icahn School of Medicine at Mount Sinai New York City New York USA; ^4^ Division of General Internal Medicine Icahn School of Medicine at Mount Sinai New York City New York USA; ^5^ Division of Pulmonary and Critical Care Medicine Icahn School of Medicine New York City New York USA; ^6^ BeiGene Ltd Emeryville California USA

**Keywords:** non‐small cell lung cancer (NSCLC), programmed cell death protein 1 (PD‐1), programmed death ligand‐1 (PD‐L1), real‐world evidence, treatment patterns

## Abstract

**Background:**

Immune checkpoint inhibitors targeting the programmed cell death protein‐1 (PD‐1) and programmed death ligand‐1 (PD‐L1) axis (collectively referred to as PD[L]1i) have demonstrated clinical benefits in non‐small cell lung cancer (NSCLC) patients. The purpose of this United States‐based real‐world study is to examine changes in the landscape of first‐line therapies for NSCLC since the introduction of PD(L)1i.

**Methods:**

Patients with NSCLC initiating first‐line treatment between May 1, 2017, and October 31, 2020, were identified in the IBM MarketScan® database. Patients were assigned groups based on first‐line therapy: PD(L)1i monotherapy, chemotherapy alone, PD(L)1i with chemotherapy, or targeted therapy for patients with actionable driver mutations.

**Results:**

A total of 5431 patients with NSCLC starting first‐line treatment were identified: chemotherapy alone 2568 (47%), PD(L)1i with chemotherapy 1364 (25%), PD(L)1i monotherapy 790 (15%), and targeted therapy 709 (13%). The use of PD(L)1i monotherapy and targeted therapy remained consistent, while the percentage of patients receiving PD(L)1i with chemotherapy more than doubled. Over a third of patients in 2019 and 2020 received chemotherapy alone. Patients aged ≥65 years (odds ratio [OR]: 0.80; 95% confidence interval [CI]: 0.68–0.95), females (OR: 0.86; 95% CI: 0.74–0.98), and those with respiratory (OR: 0.82; 95% CI: 0.71–0.94) or kidney (OR: 0.56; 95% CI: 0.40–0.77) disease were less likely to have received PD(L)1i with chemotherapy than patients that received chemotherapy alone.

**Conclusions:**

Since the approval of PD(L)1i for NSCLC, their use has significantly increased for first‐line treatment, especially when used in combination with chemotherapy. A significant proportion of patients received chemotherapy alone.

## Graphical abstract

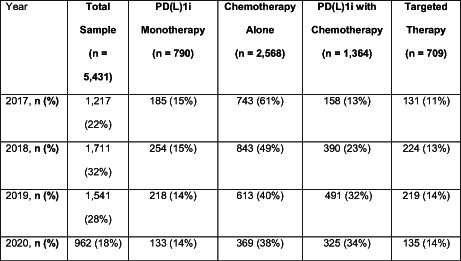

## 1 INTRODUCTION

Lung cancer is the leading cause of cancer‐related mortality worldwide with 1,796,144 deaths and 2,206,771 new cases reported in 2020.^1^ Approximately 80% to 85% of lung cancer cases are non‐small cell lung cancer (NSCLC), of which >50% of cases are diagnosed at advanced stages.^2^ For several decades, platinum‐based chemotherapy has been the standard of care for treatment of stage IV NSCLC, despite being associated with poor 5‐year survival and significant treatment‐related toxicities.^3^, ^4^ Immune checkpoint inhibitors, which target the programmed cell death protein‐1 (PD‐1) receptor/PD‐1 ligand 1 (PD‐L1) pathway in order to restore antitumor immunity have demonstrated unprecedented survival outcomes in clinical trials and are now supported by several guidelines for first‐line treatment of stage IV NSCLC.^5^

The US Food and Drug Administration (FDA) has approved the use of PD(L)1i either as monotherapy or in combination with chemotherapy for the first‐line treatment of patients with stage IV NSCLC. In 2015, pembrolizumab (PD‐1 inhibitor) was approved as monotherapy initially for high PD‐L1 expressing tumors (>50%), and then in 2019 for PD‐L1 expressing tumors (≥1%) based on the Keynote‐024 and Keynote‐042 trials, respectively.^6^ In May 2017, pembrolizumab in combination with chemotherapy received accelerated approval, for non‐squamous cell histology, regardless of PD‐L1 expression, following the findings of Keynote‐021G. Regular approval for this indication and for squamous cell carcinoma was granted in 2018 after the reporting of Keynote‐189 and Keynote‐407.^6^ Later, the IMpower110, IMpower150, and EMPOWER‐Lung 1 trials led to the approval of other immunotherapeutic agents (atezolizumab and cemiplimab, respectively) as first‐line agents.^4^, ^7^, ^8^ In 2020, nivolumab in combination with ipilimumab (anti‐CTLA4 inhibitor) was approved for NSCLC patients with PD‐L1 > 1% expression. Collectively, an increasing number of randomized controlled trials (RCTs) and real‐world data sets have demonstrated significantly prolonged overall survival (OS) and progression‐free survival (PFS), as well as more favorable side effect profiles and improved quality of life outcomes with PD(L)1i compared to platinum‐based chemotherapy.^9^, ^10^, ^11^ Therefore, all patients with NSCLC without a contraindication (e.g., autoimmune diseases) or an actionable driver mutation (e.g., epidermal growth factor receptor [EGFR], anaplastic lymphoma kinase [ALK], or c‐ros oncogene 1 [ROS1]) should receive a PD(L)1i as part of their first‐line treatment.^12^

Despite the well‐established clinical efficacy and tolerability of PD(L)1i, little is known about the current patterns of use of PD(L)1i in clinical practice as a first‐line treatment for NSCLC, either as a monotherapy or in combination with platinum‐based chemotherapy. Previous studies, conducted shortly after the approval of the first PD(L)1i in the United States and Canada, suggested that their use was increasingly being adopted; however, these studies did not extend beyond 2018.^13^, ^14^, ^15^, ^16^, ^17^ Therefore, by utilizing US‐based real‐world data from the IBM MarketScan® Research databases, this study's primary objective is to assess the temporal trends and current utilization of PD(L)1i compared to chemotherapy alone or targeted therapy (based on molecular profiling) for NSCLC treatment. In addition, the secondary objectives is to evaluate the differences in demographic and clinical characteristics that may drive treatment selection decisions in clinical practice.

## 2 MATERIALS AND METHODS

### 2.1 Patient data collection

This observational study utilized administrative claims data from the IBM MarketScan® Research database which includes the Commercial Database and the Medicare Supplemental Database.^6^ The Commercial Database contains health insurance claims for individuals with coverage from large employers who provide private healthcare coverage for employees and their families. The Medicare Supplemental Database contains claims for individuals with Medicare supplemental insurance paid for by employers. Data are de‐identified and comply with the patient confidentiality requirements of the Health Insurance Portability and Accountability Act (HIPAA). Institutional Review Board approval was not required since individual patient data were not identifiable.

Newly diagnosed patients with NSCLC were identified in the IBM MarketScan® Commercial and Medicare Supplemental databases during the study period of May 1, 2017 (the first year when PD[L]1i received approval as both monotherapy and in combination with chemotherapy for advanced NSCLC), through October 31, 2020. Eligible patients met the following criteria: (a) at least two claims within 1 year for NSCLC (ICD9: 162.x; ICD10 34.x) during the study period; (b) no NSCLC claim in the database prior to the study period; (c) continuous enrollment at least 90 days prior to the first NSCLC claim; (d) continuous enrollment for at least 60 days after first NSCLC claim; (e) ≥18 years old, (f) began NSCLC therapy with first‐line,; and (g) did not receive therapy for small cell lung cancer (claim for etoposide or irinotecan hydrochloride). For those patients that met the eligibility criteria, the date of the first NSCLC diagnosis served as the index date.

Patients were assigned to one of four groups: (1) *PD(L)1i monotherapy*: patients whose first claim was a PD(L)1i (i.e., pembrolizumab, nivolumab, atezolizumab) and had no claim for chemotherapy (i.e., carboplatin, cisplatin, paclitaxel, nab‐paclitaxel, pemetrexed, docetaxel, gemcitabine) within 45 days of diagnosis; (2) *chemotherapy alone*: first claim for chemotherapy and no PD(L)1i claim within 45 days as above; (3) *PD(L)1i with chemotherapy*: claim for a PD(L)1i and chemotherapy within 45 days, and (4) *targeted therapy*: patients that had a claim for an EGFR (i.e., osimertinib, erlotinib, afatinib, gefitinib, dacomitinib), ALK/ ROS1 (i.e., brigatinib, ceritinib, crizotinib, lorlatinib), BRAF V600E plus MEK inhibitors (i.e., dabrafenib, trametinib, vemurafenib, cobimetinib), or NTRK (i.e., larotrectinib, entrectinib) and no claim for chemotherapy or PD(L)1i within 45 days. Patients on dual check point blockade therapy with ipilimumab (anti‐CTLA4 inhibitor) and nivolumab were not included given that FDA approval occurred at the end of the study period, providing very limited data for this regimen.

We obtained pretreatment information including age, sex, insurance type, and treatment facility setting (urban or rural). We further collected clinical characteristics over the 180‐day period prior to first NSCLC diagnosis that may have influenced treatment selection, including cerebrovascular disease, congestive heart failure, diabetes, liver disease, renal disease, and COPD. The presence of the comorbidity was scored as a “1” and absence as “0.” The burden of comorbidities was assessed by calculating the Charlson comorbidity index (CCI) for each study patient over the 180 days prior to diagnosis.^18^ Higher scores on the CCI indicate a greater comorbidity burden.

### 2.2 Statistical analysis

We used descriptive statistics to examine the proportion of patients in each treatment group according to each year of the study period. Baseline differences in demographic and clinical characteristics between the study groups were tested using chi‐square, ANOVA, or Kruskal–Wallis test as indicated. We next sought to evaluate the predictors of PD(L)1i use. As decisions related to PD(L)1i monotherapy are often driven by high tumor PD‐L1 expression by immunohistochemistry (>50%), we performed a single logistic regression analysis to examine predictors of using PD(L)1i in combination with chemotherapy versus chemotherapy alone. Predictors included in the model were age, gender, urban/rural, CCI, insurance type, and year of treatment as well the presence or absence of a number of comorbidities including cerebrovascular disease, congestive heart failure, diabetes, liver disease, kidney disease, and chronic obstructive pulmonary disease or emphysema.

## 3 RESULTS

**FIGURE 1:**
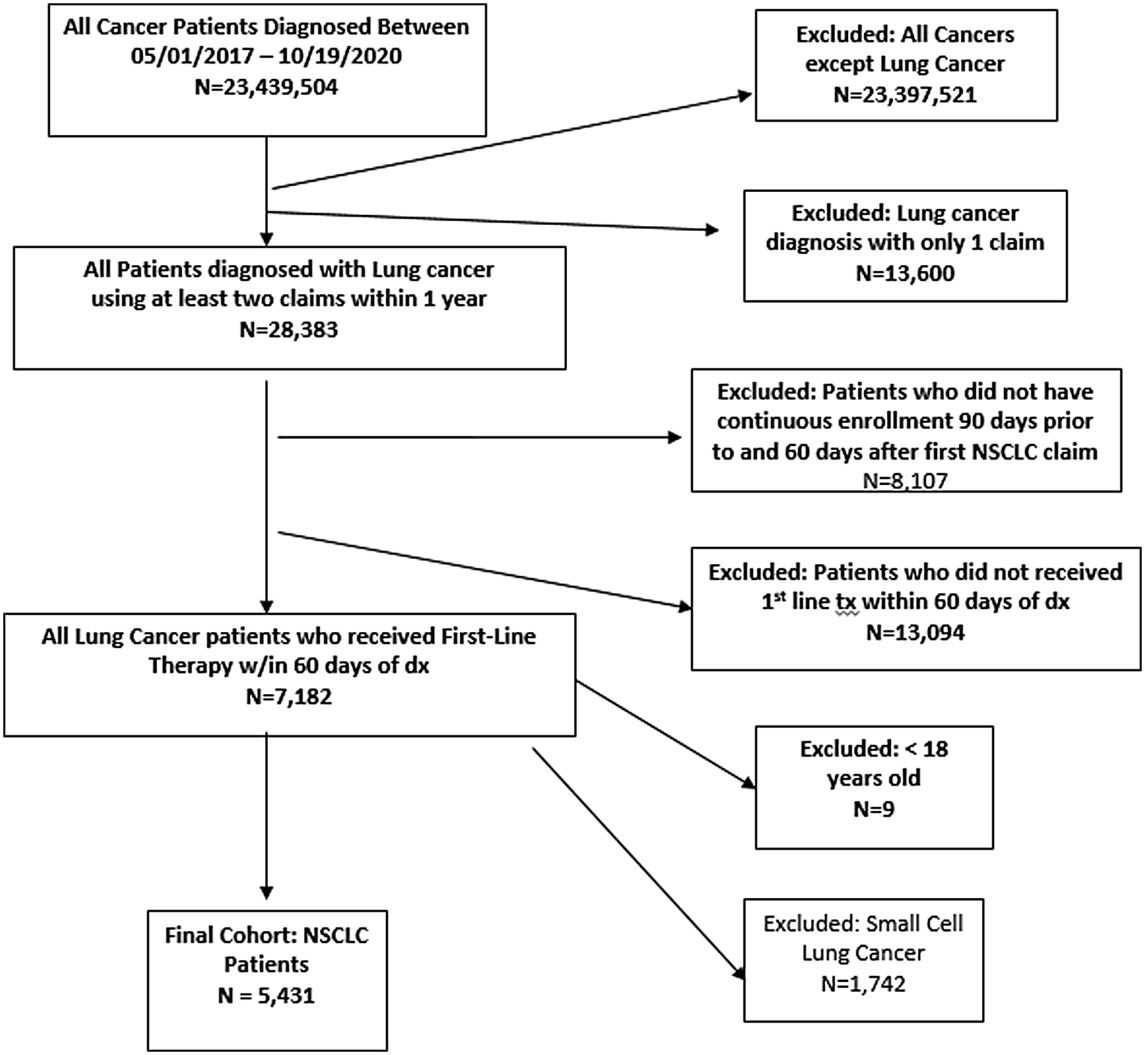
Patient selection flowchart

There were 5431 newly diagnosed patients with NSCLC starting first‐line therapy (see Figure 1). As shown in Table 1, the majority of patients between the years 2017 and 2020 received chemotherapy alone (*n* = 2568; 47%) followed by PD(L)1i with chemotherapy (*n* = 1364; 25%), PD(L)1i monotherapy (*n* = 790; 15%), and targeted therapy (*n* = 709; 13%). The proportion of patients who received PD(L)1i monotherapy and targeted therapy remained relatively stable over these 4 years. The percentage of patients receiving PD(L)1i with chemotherapy more than doubled during this same period. Finally, while the proportion of patients that received chemotherapy alone decreased by almost half over 4 years, a significant percentage of patients did not receive PD(L)1i treatment.

**TABLE 1:** Distribution of therapy groups according to year of diagnosis

Year	Total sample (*n* = 5431)	PD(L)1i monotherapy (*n* = 790)	Chemotherapy alone (*n* = 2568)	PD(L)1i with chemotherapy (*n* = 1364)	Targeted therapy (*n* = 709)
2017, *n* (%)	1217 (22%)	185 (15%)	743 (61%)	158 (13%)	131 (11%)
2018, *n* (%)	1711 (32%)	254 (15%)	843 (49%)	390 (23%)	224 (13%)
2019, *n* (%)	1541 (28%)	218 (14%)	613 (40%)	491 (32%)	219 (14%)
2020, *n* (%)	962 (18%)	133 (14%)	369 (38%)	325 (34%)	135 (14%)

### 3.1 Demographic and clinical characteristics

The PD(L)1i monotherapy and chemotherapy alone groups were older compared to those receiving combination PD(L)1i with chemotherapy and targeted therapy (*p* < 0.001; see Table 2). The distribution of sex was not similar across all four groups, with a significantly greater proportion of males receiving PD(L)1i monotherapy (55.2%) and a significantly greater proportion of females receiving targeted therapy (63.2%). Most patients received treatment in an urban setting and the distributions were similar across groups except for the targeted therapy group, which included significantly more urban patients (*p* = 0.002). There was no statistical difference in insurance type across treatment groups (*p* = 0.31); most of the patients were covered by a traditional health plan such as a health maintenance organization or preferred provider organization. The number of days from diagnosis to initiation of first‐line therapy was statistically shorter in the PD(L)1i monotherapy and targeted therapy groups than the other two chemotherapy groups (*p* < 0.001); however, the absolute difference was nominal (range: 29.69–32.58 days).

**TABLE 2:** Demographic characteristics of patients by therapy group

Characteristic	PD(L)1i monotherapy (*n* = 790)	Chemotherapy alone (*n* = 2568)	PD(L)1i with chemotherapy (*n* = 1364)	Targeted therapy (*n* = 709)	*p*‐value
Age, years, mean (SD)	63.71^a^ (12.01)	61.70^b^ (9.77)	60.48^c^ (9.14)	59.36 (11.30)	<0.001
Age group, *n* (%)					<0.001
≤64 years old	527 (66.7%)	1856 (72.3%)	1069 (78.4%)	550 (77.6%)	
≥65 years old	263 (33.3%)	712 (27.7%)	295 (21.6%)	159 (22.4%)	
Sex, *n* (%)					<0.001
Females, *n* (%)	354^a^ (44.8%)	1294^b^ (50.4%)	659^a,b^ (48.3%)	448^c^ (63.2%)	
Location of treatment, *n* (%)					0.002
Urban	685^a^ (86.7%)	2183^a^ (85.0%)	1157^a^ (84.8%)	645^b^ (91.0%)	
Rural	105^a^ (13.3%)	385^a^ (15.0%)	207^a^ (15.2%)	64^b^ (9.0%)	
Insurance, *n* (%)					0.31
Traditional health plan*	665 (84.2%)	2179 (84.9%)	1134 (83.1%)	584 (82.4%)	
Consumer‐driven plan**	125 (15.8%)	389 (15.1%)	230 (16.9%)	125 (17.6%)	
Days from diagnosis to therapy initiation, mean (SD)	30.48^a^ (16.36)	32.58^b^ (15.11)	32.04^b^ (14.42)	29.69^a^ (12.24)	<0.001
CCI, mean (SD)	7.23^a^ (3.06)	5.95^b^ (3.73)	6.79^c^ (2.76)	6.81^c^ (2.65)	<0.001
Cerebrovascular disease, *n* (%)	122 (15.4%)	308 (12.0%)	180 (13.2%)	88 (12.4%)	0.08
Congestive heart failure, *n* (%)	72^a^ (9.1%)	187^a,b^ (7.3%)	87^b^ (6.4%)	29^c^ (4.1%)	0.001
Diabetes, *n* (%)	132^a,b^ (16.7%)	471^a^ (18.3%)	202^a,b^ (14.8%)	95^a^ (13.4%)	0.01
Liver disease, *n* (%)	117^a,b^ (14.8%)	316^b^ (12.3%)	195^a,b^ (14.3%)	124^a^ (17.5%)	0.003
Kidney disease, *n* (%)	81^a^ (10.3%)	171^b^ (6.7%)	57^c^ (4.2%)	26^c^ (3.7%)	<0.001
COPD or emphysema, *n* (%)	287^a^ (36.3%)	1202^b^ (46.8%)	545^a^ (40.0%)	67^c^ (9.4%)	<0.001

*Note*: ^a,b,c^ Within a row, values with different superscripts are significantly different at *p* < 0.05 level.

* Traditional Health Plan = preferred provider organization, health maintenance organization, comprehensive, point of service plan w/wo cap, exclusive provider organization.

** Consumer‐Driven Plans, consumer‐driven health plan; high deductible health plan. CCI, Charlson Comorbidity Index; COPD, Chronic Obstructive Pulmonary Disease; SD, Standard Deviation; PD(L)1i, PD1/PD‐L1 inhibitor.

The distribution of comorbidities was different according to which first‐line treatment regimen patients received (see Table 2). Overall, the PD(L)1i monotherapy group had the highest CCI score (mean = 7.23; SD = 3.06) while the chemotherapy alone group had the lowest (mean = 5.95; SD = 3.73; *p* < 0.001). The proportion of patients with congestive heart failure was significantly higher in the PD(L)1i monotherapy group compared to the PD(L)1i with the chemotherapy group. Diabetes and COPD were more prevalent among the chemotherapy alone group than the other therapy groups. The proportion of patients with kidney disease was higher in the PD(L)1i monotherapy and chemotherapy alone groups than the other two therapy groups.

### 3.2 Predictors of treatment selection between combination PD(L)1i with chemotherapy versus chemotherapy alone in patients with NSCLC

While PD(L)1i monotherapy is primarily driven by PD‐L1 expression, predictors of combining PD(L)1i with chemotherapy compared to chemotherapy alone are less clear. In adjusted analyses, older patients (≥65 years of age; odds ratio [OR]: 0.80; 95% CI: 0.68–0.95), females (OR: 0.86; 95% CI: 0.74–0.98), and patients with renal disease (OR: 0.56; 95% CI: 0.40–0.77) and COPD (OR: 0.82; 95% CI: 0.71–0.94) were less likely to have received combination PD(L)1i with chemotherapy than patients who received chemotherapy without a PD(L)1i (Table 3). A higher CCI score (OR: 1.08; 95% CI: 1.06–1.11) and those treated in later years following approval (OR: 2.22, 95% CI: 1.80–2.75; OR: 3.87, 95% CI: 3.14–4.79; OR: 4.17, 95% CI: 3.31–5.25 in 2018, 2019, and 2020, respectively, relative to patients in 2017) were more likely to be treated with combination PD(L)1i with chemotherapy compared to chemotherapy without a PD(L)1i.

**TABLE 3:** Factors Predicting use of combination PD(L)1i with chemotherapy versus chemotherapy alone

Characteristics	OR	95% CI
Age Group (1 = ≥65; 0 = ≤64)	0.80	0.68–0.95
Gender (1 = female; 0 = male)	0.86	0.74–0.98
Urban/Rural (Urban = 1; Rural = 0)	0.98	0.81–1.19
CCI	1.08	1.06–1.11
Insurance type (1 = Traditional; 2 = Consumer driven)	1.03	0.85–1.24
Cerebrovascular disease	1.08	0.88–1.33
Congestive heart failure	0.85	0.64–1.12
Diabetes	0.86	0.72–1.04
Liver disease	1.00	0.82–1.23
Kidney disease	0.56	0.40–0.77
COPD or Emphysema	0.82	0.71–0.94
Year of treatment (in relation to 2017)		
2018	2.22	1.80–2.75
2019	3.87	3.14–4.79
2020	4.17	3.31–5.25

*Note*: Chemotherapy w/ PD(L)1i = 1; Chemotherapy w/o PD(L)1i = 0.Abbreviations: CCI, Charlson Comorbidity Index; COPD, Chronic Obstructive Pulmonary Disease.

## 4 DISCUSSION

The current study is among the first to examine the real‐world utilization of first‐line therapies in patients with NSCLC since the approval of PD(L)1i as monotherapy or in combination with chemotherapy. We found that usage of PD(L)1i monotherapy and targeted therapies have remained unchanged, while PD(L)1i combined with chemotherapy has more than doubled during the 4‐year study period. Notably, while the use of chemotherapy without PD(L)1i has declined significantly, over a third of potentially eligible patients still did not receive PD(L)1i in 2019 and 2020. In the adjusted analysis, we further found that patients who receive chemotherapy without PD(L)1i are more likely to be older, female, and have renal and/or pulmonary comorbidities. Our findings provide important information for researchers, clinicians, and policy makers regarding the adoption trends of immunotherapy and highlight a substantial subset of patients not receiving these novel agents that are deserving of increased focus to ensure optimal management.

Despite the well‐established clinical efficacy and tolerability of PD(L)1i, there may be several concerns for the universal adoption of these novel agents. Rapid approvals of new drugs given “breakthrough” designation by the FDA have allowed for accelerated development and early patient access to cancer therapeutics. However, many oncologists may be hesitant to adopt new drugs based on the following reasons: (1) endpoints used for approval only provide preliminary evidence of efficacy (i.e., response rates, 1‐year OS); (2) lack of generalizability to patients with cancer treated in real‐world practices; and (3) lack of long‐term data regarding side effects. As such, a few prior studies have demonstrated that the extent of uptake of PD(L)1i in clinical practice has been suboptimal for several cancer types.^16^ In fact, one early study reported that shortly after FDA approval, only 60% of eligible patients with cancer (e.g., melanoma, NSCLC, or renal cell carcinoma) had received PD‐1 agents (e.g., nivolumab or pembrolizumab).^16^ To date, no studies have evaluated changes in practice patterns of PD(L)1i use for lung cancer over more recent years. While it might be expected that use of PD(L)1i will increase with time and familiarity, we found that similar percentages of patients received PD(L)1i monotherapy each year throughout the study period.

Since FDA approval in 2015, PD(L)1i monotherapy has been typically used for patients with NSCLC that have high tumor expression of PD‐L1 tumors (≥50%). Several prior studies have shown that approximately 28% of NSCLC fall under this subgroup.^10^, ^19^ Yet, the proportion of patients receiving PD(L)1i monotherapy each year in our real‐world study remained consistent around 16%–17% (excluding targeted therapy patients). This stability may be due to the fact that treatment decisions regarding PD(L)1i monotherapy in clinical practice are primarily driven by tumor expression of PD‐L1, with high (>50%) PD‐L1 expression receiving pembrolizumab alone as first line treatment. We would not expect the proportion of high PD‐L1 expressing tumors, and therefore the indication for PD(L)1i monotherapy, to change during the study period. Furthermore, we did not find increased use of PD(L)1i monotherapy after the approval for PD(L)1i monotherapy was expanded to include all NSCLCs with PD‐L1 expression (≥1%). When evaluating demographic and clinical characteristics of each treatment group, patients that received PD(L)1i monotherapy were older and had higher comorbidity scores than the other groups, likely reflecting the increased tolerability compared to cytotoxic chemotherapy. Additionally, a higher proportion of males to females received PD(L)1i monotherapy use—a possible explanation may be that higher smoking rates in males are associated with higher tumor mutation burden and increased PD‐L1 expression.^20^, ^21^

Following the approval of PD(L)1i monotherapy, PD(L)1i with chemotherapy was approved for first‐line treatment of patients with NSCLC regardless of PD‐L1 expression. As a result, we observed a significant shift of practice pattern to combine PD(L)1i with chemotherapy. Yet, a significant proportion of patients only received chemotherapy alone. Our analysis examining predictors of PD(L)1i use in combination with chemotherapy indicated that older patients were less likely to have a PD(L)1i added in combination with chemotherapy. While there may be concerns of tolerability for older patients, pooled analysis of clinical trials has demonstrated that adding PD(L)1i to chemotherapy did not increase toxicity rates compared to chemotherapy alone.^22^ However, there were increased rates of immune‐related toxicities with the combination that may be responsible for our findings of lower PD(L)1i use in patients with respiratory (i.e., COPD or emphysema) and renal disease, regardless of their overall comorbidity burden. With increased familiarity with these agents, treating physicians may be becoming more comfortable with managing the immune‐related toxicities as demonstrated by the increasing use of PD(L)1i in more recent years.

Whereas the real‐world results of this retrospective analysis provide important insight into patterns of PD(L)1i use, they should be considered alongside some limitations of the study. First, this observational analysis utilized retrospective administrative claims data, which lack potentially important clinical details for the reasons of therapy selection (e.g., race/ethnicity, biomarkers, the number and location of metastatic lesions, the type of clinical practice [academic vs. community]). Despite this, our study was still able to evaluate the associations and impact of several demographics (i.e., age, sex) and individual comorbidities on treatment patterns. Second, due to the lack of granularity in ICD coding, information pertaining to the staging is not available. However, the treatment groups were defined using regimens for advanced NSCLC according to both FDA approvals and NCCN guidelines (with the exception of pembrolizumab's indication for unresectable stage III disease in patients who cannot tolerate definitive chemoradiation). Third, this study was limited to only those individuals with commercial health coverage or private Medicare supplemental coverage. Consequently, results of this analysis may not be generalizable to patients with NSCLC with other insurance or without health insurance coverage, or patients outside the United States. Finally, the proportion of patients over 65 years old in the MarketScan® database is lower than what is typically seen in the general lung cancer population and therefore may not comprehensively reflect the treatment patterns of older NSCLC patients. Despite these limitations, this study still provides important and up‐to‐date real‐world data describing practice patterns of PD(L)1i use for first‐line treatment of stage IV NSCLC.

## 5 CONCLUSION

The current study highlights that PD(L)1i therapy utilization in patients with NSCLC has steadily grown in the United States since the approval of the first PD(L)1i in 2015. Moreover, this study identified important demographic (e.g., age and sex) and clinical (e.g., renal and pulmonary comorbidities) factors associated with the use of PD(L)1i in NSCLC treatment. Taken together, our findings demonstrate that while the adoption of PD(L)1i is increasing, a significant proportion of patients still do not receive PD(L)1i. Further qualitative research should be undertaken to elucidate the underlying reasons of slow adoption. As more immunotherapy agents and combinations are approved and used in clinical practice, practice patterns should continue to be evaluated to ensure NSCLC patients are receiving optimal treatment.

## CONFLICT OF INTEREST

RS, DH, BT, and BB are employees of and own stock in Beigene LTD. FRH reports the following conflict of interest: Bristol‐Myers Squibb, Genentech, AstraZeneca/Daiichi, Regeneron, Novartis, Sanofi‐Genzyme, Merck, OncoCyte, and Amgen. RRV has served on advisory boards for Bristol‐Myers Squibb, AstraZeneca, Merck, Novocure, on unbranded speaker's bureau of AstraZeneca, received consulting honorarium from Beigene and Onconova Therapeutics, and research grants from BMS, Astrazeneca, and EMD Serono. JW received consulting honorarium from Sanofi, GSK, and Banook and a research grant from Sanofi. JW reports consulting honorarium from Sanofi, Atea Pharmaceutical, and Banook and grants from Sanofi, Regeneron, and Arnold Consultants. ET reports no conflicts. Editorial assistance was provided by Jason C. Allaire, PhD of Generativity ‐ Health Economics and Outcomes Research, Durham, NC.

## AUTHOR CONTRIBUTION

Veluswamy contributed conceptualization, methodology, writing of original draft, and reviewing and editing. Hirsch, Taioli and Wisnivesky contributed to the interpretation of results, writing of original draft, and reviewing and editing. Strauss, Harrough, and Tang contributed conceptualization, methodology, writing of original draft, and reviewing and editing. Barnes contributed to conceptualization, methodology, writing of original draft, supervision, and reviewing and editing. All authors reviewed and approved the final version for submission.

## ETHICS STATEMENT

Institutional review board approval was not required as MarketScan data are recorded in such a manner that subjects cannot be identified, directly or through identifiers linked to the subjects. Meeting these conditions makes this research exempt from the requirements of 45 CFR 46.101 under the Department of Health and Human Services.

## Data Availability

Data subject to third party restrictions The data that support the findings of this study are available from IBM Watson Health. Restrictions apply to the availability of these data, which were used under license for this study. Data are available from the authors with the permission of IBM Watson Health.
